# Reduced port laparoscopic surgery using umbilical zigzag incision for Meckel’s diverticulitis

**DOI:** 10.1016/j.ijscr.2020.12.033

**Published:** 2020-12-24

**Authors:** Ippei Yamana, Jun Ohishi, Tatsuya Hashimoto, Hiroki Tani, Yasuhide Fuchino, Hiroshi Ohtani, Suguru Hasegawa

**Affiliations:** a Department of Surgery, Hakujyuji Hospital, 3-2-1, Ishimaru, Nishi-ku, Fukuoka, 819-8511, Japan; b Department of Pathology, Hakujyuji Hospital, 3-2-1, Ishimaru, Nishi-ku, Fukuoka, 819-8511, Japan; c Department of Gastroenterological Surgery, Fukuoka University School of Medicine, 7-45-1, Nanakuma, Jonan-ku, Fukuoka, 814-0180, Japan

**Keywords:** Reduced port laparoscopic surgery, Zigzag incision, Meckel’s diverticulitis

## Abstract

**Introduction:**

Reduced port laparoscopic surgery using an umbilical zigzag incision is comparable to conventional multiport laparoscopic surgery. This method is associated with improved cosmesis and decreased wound pain.

**Presentation of case:**

A 67-year-old man visited our hospital. He presented emergency room with a chief complaint of right lower abdominal pain by walking. The patient was diagnosed Meckel’s diverticulitis by computed tomography (CT). At first, antibiotics therapy and fasting were performed. Three months later, the patient underwent resection of Meckel’ diverticulum with zigzag transumbilical laparoscopic surgery. The patient’s postoperative course was good, and he rarely felt wound pain. The patient started oral intake three days after surgery, and was discharged 10 days after surgery.

**Discussion:**

Zigzag transumbilical laparoscopic surgery is very useful for resection of the small intestine. This method is associated with improved cosmesis and decreased wound pain. In the case of Meckel’s diverticulitis, we suggest that interval resection of Meckel’s diverticulum was very useful as it was an operation that could be performed easily.

**Conclusion:**

Reduced port laparoscopic surgery using an umbilical zigzag incision is considered to be an excellent technique in terms of operability and aesthetic outcomes.

## Highlights

• In our case report, we suggested that reduced port laparoscopic surgery using an umbilical zigzag incision is comparable to conventional multiport laparoscopic surgery.
• This method is associated with improved cosmesis and decreased wound pain.
• We herein report a case involving interval resection of Meckel’s diverticulum using a transumbilical approach with a zigzag incision, which was performed three months after the appearance of symptoms.

## 1 Introduction

Meckel’s diverticulum is a common anomaly of the gastrointestinal tract. Most patients with Meckel’s diverticulum are asymptomatic; however, some patients develop bleeding, inflammation, and perforation, which requires surgical treatment.

Recently, reduced port surgery has become widespread in many fields of laparoscopic surgery. Hachisuka et al. applied a transumbilical approach with zigzag incision in 2012 [1]. A zigzag skin incision enables the enlargement of the fascial and peritoneal incisions. We suggest that this method would be feasible and beneficial for concomitant laparoscopic surgery.

We herein report a case involving interval resection of Meckel’s diverticulum using a transumbilical approach with a zigzag incision, which was performed three months after the appearance of symptoms with a literature review in line with the SCARE criteria [2].

## 2 Presentation of the case

**Fig. 1:**
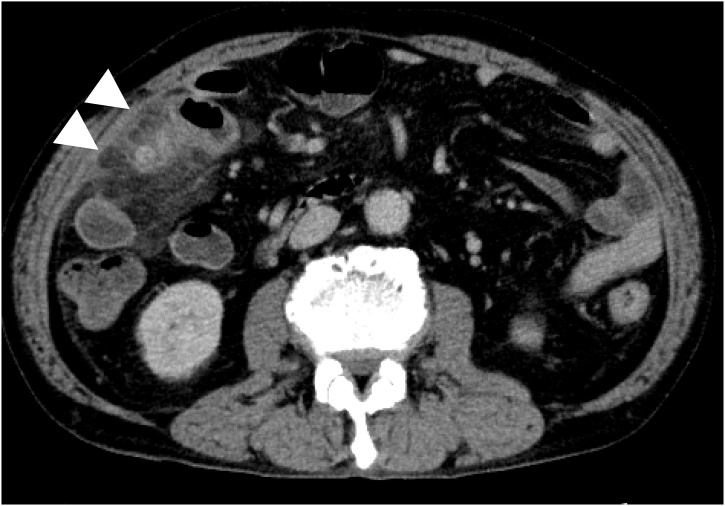
CT showed an enhanced mass of 20 mm diameter that continued to the wall of ileum. A fat-dense area was enhanced around the mass.

**Fig. 2:**
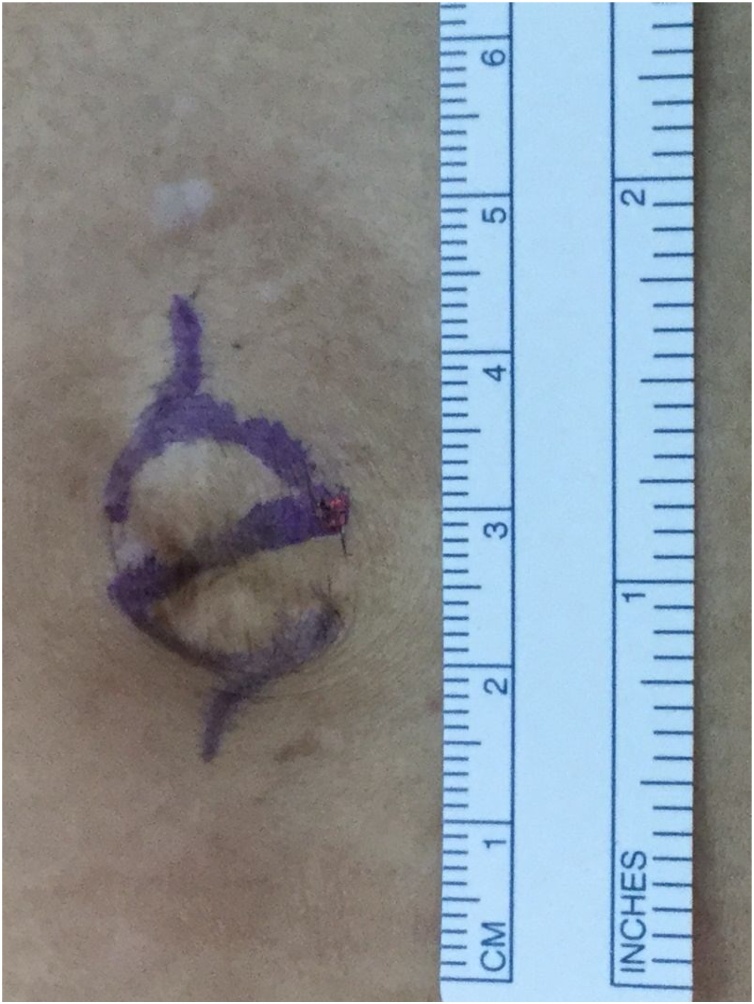
A 3-cm zigzag incision was made at the umbilicus.

**Fig. 3:**
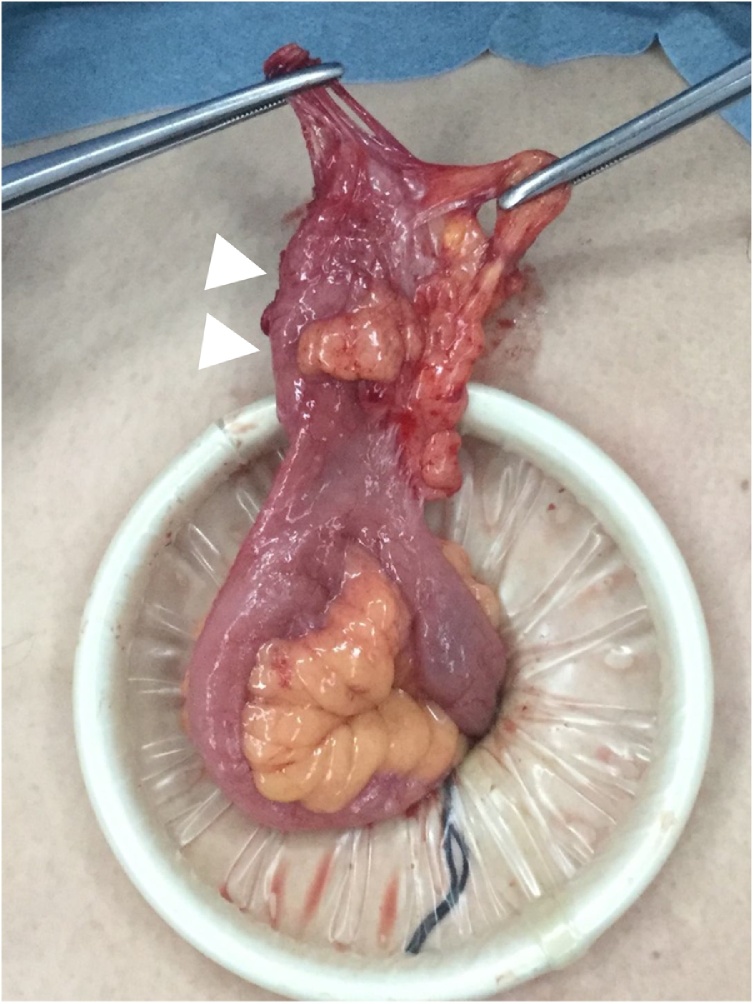
A diverticulum of 50 mm in diameter was recognized was recognized 30 cm proximal to the terminal ileum.

**Fig. 4:**
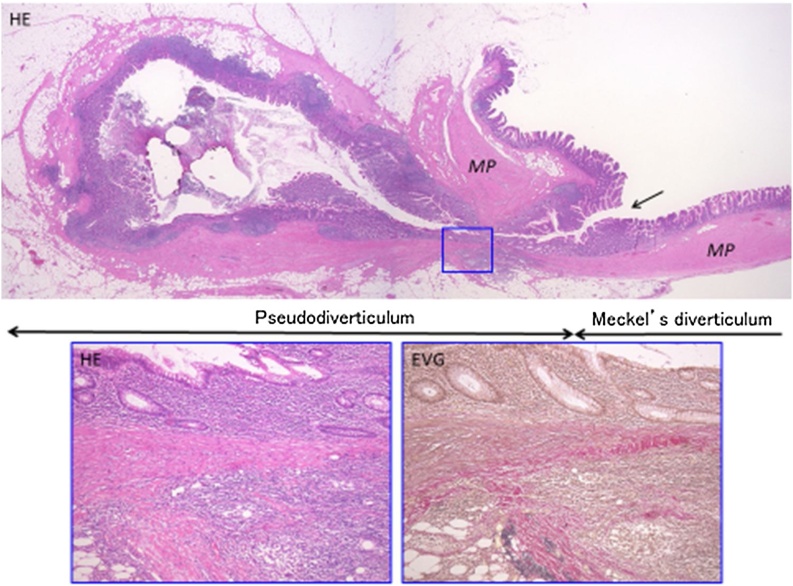
Microscopic appearance. A loupe view of the resected Meckel’s diverticulum shows almost normal ileal wall with well-formed villi and the muscularis propria (right side of upper picture). The tip revealed a mucosal herniation (arrow) through the diverticular wall, outpouching in the mesentery. This outpouching area showed poorly-developed muscularis mucosa and lacked the muscularis propria (left side of upper picture). The mucosa shows erosions, villous atrophy, and lymphoid hyperplasia, surrounded with marked fibroelastosis. Lower pictures stained with hematoxylin & eosin (HE, left) and Elastica van Gieson (EVG, right) are higher magnifications of the square area of upper picture.

**Fig. 5:**
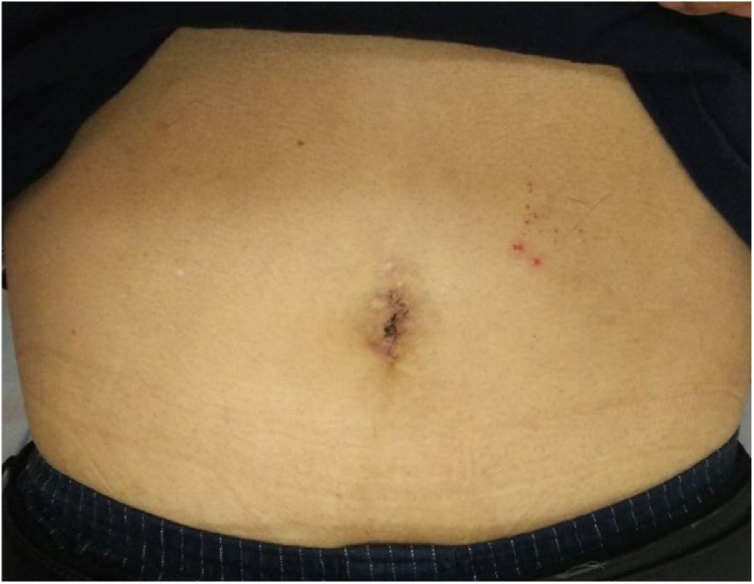
One month later after discharge, the umbilical portion showed scarring.

The patient was a 67-year-old man who presented to our hospital with a chief complaint of right lower abdominal pain by walking. The patient’s past history included hypertension and hyperuricemia. The patient suffered from Blumberg’s sign on the right lower side. Laboratory data showed that his inflammatory reaction level was increased. CT showed an enhanced mass of 20 mm in diameter that continued to the wall of the ileum and a fat–dense area was enhanced around the mass (Fig. 1). The patient was diagnosed with Meckel’s diverticulitis. At first, antibiotic therapy and fasting was performed. The patient subsequently recovered and oral intake was started. A lower digestive tract examination was performed, revealed no signs of the tumor. Three months later, interval resection of Meckel’s diverticulum was performed by transumbilical laparoscopic surgery with a zigzag incision. This operation was performed by specialist of gastrointestinal surgery. A 3-cm zigzag incision was made at the umbilicus (Fig. 2). Then, a 6-cm incision was made at the fascia. A Gel-POINT access platform (Applied Medical, CA, Japan) was inserted through the wound. Three ports were used, one for the scope and two for handling forceps. Almost no adhesion was observed in the abdominal cavity. A diverticulum of 50 mm in diameter was recognized on the antimesenteric side of 30 cm proximal to the terminal ileum. There were no other abdominal lesions in the remaining intestine. It was easy to elevate the diverticulum outside of the abdomen (Fig. 3). The vitelline artery and vein were identified and ligated. Meckel’s diverticulum was resected by endo GIA (camel, 45 mm; Covidien, MA, Japan). Examination of the resected specimen revealed that the mucosa was intact. Pathological examination of the resected Meckel’s diverticulum were covered by almost normal ileal mucosa with villous formation. There was well-developed muscularis propria (MP), however the tip revealed a mucosal herniation (Fig. 4 arrow) through the diverticular wall, outpouching in the mesentery. This outpouching area showed poorly-developed muscularis mucosa and lacked the muscularis propria, identical to pseudodiverticulum (Fig. 4). It contained mixed red blood cells, neutrophils and numerous colonies of bacteria in the lumen and covered with ileal-type mucosa showing erosions, villous atrophy, and lymphoid hyperplasia. Furthermore, the wall showed marked fibroelastosis, consistent with scarred stage of diverticulitis (Fig. 4). The patient’s postoperative course was good, and he rarely felt wound pain. Oral intake was started three days after surgery, and the patient was discharged 10 days after surgery. At one month later after discharge, scarring of the umbilical portion was observed (Fig. 5).

## 3 Discussion

Meckel’s diverticulum is a congenital true diverticulum composed of residual tissue of the fetal yolk duct. The name is derived from the German anatomist Johann Friedrich Meckel who described this entity in the early nineteenth century [3]. The prevalence of Meckel’s diverticulum is reported to be 0.6–4% in the general population [4]. In previous reports, bleeding, diverticulitis, intestinal obstruction, invagination, or perforation accounted for 4–16% of all cases of Meckel’s diverticulum [4]. Hansen et al. reported a systematic review of 92 articles on Meckel’s diverticulum that were reported in the 21st century. Meckel’s diverticulum is located at a mean of 52.4 cm proximal to the ileocecal valve, the mean length is 3.05 cm, and the mean diameter is 1.58 cm. Approximately 4–9% of patients present with symptoms [5].

Single port laparoscopic surgery with a transumbilical approach and a zigzag incision was first described by Hachisuka et al. [1]. Since then, various cases have been reported. Kato et al. reported two cases in which single-stage laparoscopic surgery was performed for bilateral organ tumors using a transumbilical approach with a zigzag incision [6]. Umeda et al. reported 5 cases in which reduced port endo laparoscopic surgery was performed using an umbilical zigzag incision for cholecystectomy, partial gastrectomy, cecal wedge resection, left colectomy, sigmoidectomy, low anterior resection, right colectomy [7]. Kaneko et al. reported 2 cases in which reduced port laparoscopic radical nephrectomy was performed using an umbilical zigzag skin incision for renal cell carcinoma [8].

A 3-cm umbilical zigzag incision in laparoscopic surgery enables the incision to create a larger fascial incision (at most 6 cm) and peritoneal opening. We considered that for a specimen of <6 cm in size, it is possible to elevate the resected specimen, easily. Furthermore, by using a single port with multiple working channels, this method can reduce the number of incision and improve cosmesis and the rate of incisional hernia and port site-related complications.

On the other hand, an umbilical zigzag incision in laparoscopic surgery may potentially include some problems. Kato et al. suggested that an additional incision was required for cases with larger specimens [6]. However, after a few months, incised wound was less noticeable in comparison to cases without an additional incision. We considered that an additional incision should be allowed in cases involving a specimen larger than 6 cm. Surgery should be carefully performed to avoid perforation, especially in malignant cases.

In our case, interval resection of Meckel’s diverticulectomy was performed three months after the patient’s first admission. Recently, interval appendectomy is often used for the treatment of acute appendicitis, especially in cases of complicated appendicitis. Simillis et al. reported a meta-analysis of 17 studies that reported on 1,572 patients with appendicitis. In their report, 847 patients received conservative treatment and 725 underwent acute appendectomy [9]. Conservative treatment was associated with significantly lower rates of overall complications, wound infection, abdominal pelvic abscess, bowel obstruction, and reoperation. In our case, in the abdominal cavity, less adhesion was recognized and we were able to perform the operation easily and safety. Pathologically, Meckel’s diverticulum consisted of almost normal ileal wall and revealed scarred diverticulitis of secondary pseudodiverticulum arising at the tip of Meckel’s diverticulum. There was scattered lymphoplasmacytic infiltration but no active inflammation was present in the wall. We recommended interval resection of Meckel’s diverticulum after the scarred stage, 3 months after the appearance of symptom. We also consider that one of the benefits is that it allows a lower digestive tract examination to be performed before the operation. If a malignant tumor is recognized in the lower digestive tract, the operation method should be considered. We consider that the interval resection of Meckel’s diverticulum allows for a safe operation, with a decreased risk of complication, similarly to interval appendectomy.

Reduced port laparoscopic surgery using an umbilical zigzag incision is considered to be an excellent technique in terms of operability and aesthetic outcomes. A larger clinical trial of this surgical technique with quantitative evaluations, such as the pain score and the patient’s satisfaction score is needed in the future.

## Declaration of Competing Interest

The authors report no declarations of interest.

## Funding

Sources of funding is nothing.

## Ethical approval

Ethical approval has been given.

## Consent

A patients in case report was given Informed consent.

## Author’s contribution

This paper is case report.

## Registration of research studies

Not Applicable.

## Guarantor

Suguru Hasegawa Professor.

## Provenance and peer review

Not commissioned, externally peer-reviewed.

## References

[1] Hachisuka T., Kinoshita T., Yamakawa T., Kurata N., Tsutsuyama M., Umeda S., Tokunaga S., Yarita A., Shibata M., Shimizu D., Shikano T., Hattori K., Mori T., Shinohara M., Miyauchi M. (2012). Transumbilical laparoscopic surgery using GelPort through an umbilical zigzag skin incision. Asian J. Endosc. Surg..

[2] Agha R.A., Borrelli M.R., Farwana R., Koshy K., Fowler A., Orgill D.P., For the SCARE Group (2018). The SCARE 2018 statement: updating consensus Surgical CAse REport (SCARE) guidelines. Int. J. Surg..

[3] Meckel J.F. (1809). Uber die Divertikel am Darmkanal. Arch. Physiol..

[4] Sagar J., Kumar V., Shah D.K. (2006). Meckel’s diverticulum: a systematic review. J. R. Soc. Med..

[5] Hansen C.C., Søreide K. (2018). Systematic review of epidemiology, presentation, and management of Meckel’s diverticulum in the 21st century. Medicine (Baltimore).

[6] Kato Y., Kato R., Takayama M., Ikarashi D., Onoda M., Matsuura T., Kanehira M., Takata R., Baba S., Kimura T., Otsuka K., Sugimura J., Omori S., Sasaki A., Obara W. (2018). Single-stage laparoscopic surgery for bilateral organ tumors using a transumbilical approach with a zigzag incision: a report of two cases. BMC Urol..

[7] Umeda S., Hachisuka T., Otsu T., Hishida M., Nagai S., Shimizu M., Kobayashi H., Nozaki H. (2018). Reduced-port endo-laparoscopic surgery using umbilical zigzag incision for concomitant operations: a case series. Int. J. Surg. Case Rep..

[8] Kaneko G., Shirotake S., Kanao K., Oyama M. (2020). Reduced port laparoscopic radical nephrectomy using an umbilical zigzag skin incision for renal cell carcinoma. Int. Cancer Conf. J..

[9] Simillis C., Symeonides P., Shorthouse A.J., Tekkis P.P. (2010). A meta-analysis comparing conservative treatment versus acute appendectomy for complicated appendicitis (abscess or phlegmon). Surgery.

